# Fibrinogen and clot-related phenotypes determined by fibrinogen polymorphisms: Independent and IL-6-interactive associations

**DOI:** 10.1371/journal.pone.0187712

**Published:** 2017-11-03

**Authors:** H. Toinét Cronjé, Cornelie Nienaber-Rousseau, Lizelle Zandberg, Zelda de Lange, Fiona R. Green, Marlien Pieters

**Affiliations:** 1 Centre of Excellence for Nutrition, North-West University, Potchefstroom, South Africa; 2 Division of Cardiovascular Sciences, School of Medical Sciences, Faculty of Biology, Medicine & Health, University of Manchester, Manchester, United Kingdom; Institut d'Investigacions Biomediques de Barcelona, SPAIN

## Abstract

Interleukin-6 (IL-6) induces the expression of fibrinogen, and polymorphic variation within the fibrinogen genes is believed to alter the magnitude of this expression. The identification of the functional relevance of individual fibrinogen single nucleotide polymorphisms (SNPs) has been hindered by the high linkage disequilibrium (LD) reported in the European fibrinogen gene locus. This study investigated two novel and 12 known fibrinogen SNPs of potential functional relevance, in 2010 Tswana individuals known to have low LD. We aimed to identify functional polymorphisms that contribute to clot-related phenotypes and total and γ’ fibrinogen concentrations independently and through their interaction with IL-6, by taking advantage of the high fibrinogen and IL-6 concentrations and the low LD reported in black South Africans. Fibrinogen was significantly associated with IL-6, thereby mediating associations of IL-6 with clot formation and structure, although attenuating the association of IL-6 with clot lysis time. None of the common European fibrinogen haplotypes was present in this study population. Putative functional fibrinogen SNPs *FGB*–rs7439150, rs1800789 (–1420_*G/A*_) and rs1800787 (–148_*C/T*_*)* were significantly associated with fibrinogen concentration and altered clot properties, with several associations significantly influenced by IL-6 concentrations. The impact of harbouring several minor fibrinogen SNP alleles on the association of IL-6 and fibrinogen concentration was cumulative, with possession of each additional minor allele showing a stronger relationship of IL-6 with fibrinogen. This was also reflected in differences in clot properties, suggesting potential clinical relevance. Therefore, when investigating the effect of fibrinogen genetics on fibrinogen concentrations and CVD outcome, the possible interactions with modulating factors and the fact that SNP effects seem to be additive should be taken into account.

## Introduction

Fibrinogen is central to blood coagulation and the inflammatory response. As a haemostatic protein, fibrinogen is activated by thrombin to form fibrin, the main constituent of a blood clot [1]. As an acute phase reactant, fibrinogen is up-regulated upon stimulation by inflammatory cytokines, primarily interleukin-6 (IL-6), in response to physiological trauma such as infection/inflammation [2]. Both fibrinogen and IL-6 are prospectively associated with cardiovascular disease (CVD) risk [3–5], although causality is under debate [6–9].

The fibrinogen phenotype is heritable and several single nucleotide polymorphisms (SNPs) within the fibrinogen α, β and γ chain genes (*FGA*, *FGB*, *FGG*) have been identified as contributors to this heritability in Europeans [10–13]. Fibrinogen expression is regulated on two levels: under basal conditions and during the acute phase response [14, 15]. This acute phase-induced increase in fibrinogen is largely mediated by IL-6, through the JAK/STAT pathway. In addition, sequences responsive to IL-6, and crucial for full IL-6-induced fibrinogen expression, have been identified upstream of the fibrinogen genes [14, 15].

Genetic variation in the fibrinogen genes is hypothesised to alter the magnitude of fibrinogen expression in response to IL-6 [13, 16, 17]. Thus far, the focus of investigations has been on polymorphisms within the *FGB* promoter region that have been implicated in both epidemiological [16] and *in vitro* [17] studies to interact with IL-6 in influencing fibrinogen concentrations. Greater IL-6-induced fibrinogen expression has been reported for the *FGB* rs1800790_*A*_ (*–*455_*A*_) and rs1800791_*A*_ (–854_*A*_) [16–20], while the opposite has been observed for *FGB* rs1800789 (–1420_*A*_) and rs1800787 (–148_*T*_) [16, 17]. Apart from the *FGB* polymorphisms, IL-6 interacted with *FGA*-rs2070011_*T*_ *in vivo* to enhance fibrinogen expression, whereas *FGA*-rs6050, *FGG*-rs2066865 and *FGG*-rs1049636 had no effect [13].

In addition to regulating total fibrinogen concentration, IL-6 is able to up-regulate the production of γ’ fibrinogen, a common splice variant, comprising 8 to 15% of total fibrinogen concentration [21–23]. IL-6 specific responsive elements have been characterised in the *FGG* promoter region [24, 25], and *in vitro* research reported greater increases in γ’ compared to total fibrinogen in the presence of IL-6, suggesting alterations to the alternative splicing of the γ fibrinogen gene during inflammation [22]. Elevated fibrinogen γ’ concentration is a risk factor for arterial thrombosis, although research on the individual and IL-6-interactive effects of fibrinogen polymorphisms predicting γ’ fibrinogen concentrations is limited [26–29]. Increased IL-6, total and γ’ fibrinogen are also independently associated with altered clot properties, including faster clot formation, increased fibrin density, thinner fibrin fibres and decreased clot permeability [30–34]. The functional effects of the interactions between genetic variants and IL-6 on clot properties have not been described previously.

Tight LD within the fibrinogen SNPs in Europeans has made the identification of functional SNPs difficult. Studies have implicated the *FGB* -455_*G/A*_, -854_*G/A*_ and -148_*C/T*_ polymorphisms to be functional in Europeans, although findings from epidemiological [16] and experimental work [17] have been contradictory. The African population is known for its complex LD pattern and great genetic diversity, more so than any other population [35, 36]. Furthermore, increased fibrinogen and IL-6 concentrations have been observed in black South Africans when compared to their white counterparts [37, 38]. The South African arm of the prospective urban and rural epidemiology (PURE) study indicated low LD together with markers indicative of chronic low-grade inflammation—as reported by increased fibrinogen, IL-6, homocysteine and C-reactive protein [39, 40]—making this cohort ideal for the investigation of independent and IL-6-regulated gene interactions. Therefore, this study investigates selected fibrinogen polymorphisms in terms of their independent and IL-6-interactive associations with total and γ’ fibrinogen concentrations, and the downstream functional effects in terms of clot formation, structure and lysis utilizing the unique Tswana population. Our results contribute to the broader understanding of potential factors that regulate fibrinogen-related, clinically relevant phenotypes.

## Materials and methods

### Study population and ethical considerations

This study is affiliated with the international PURE [41] study and is a cross-sectional investigation of the baseline data collected in South Africa. Details pertaining to participant selection and recruitment were published previously [39]. In short, the study population consisted of 2010 apparently healthy, self-identified black Tswana-speaking South African adults between the ages of 35 and 70 years. Participants provided voluntary written informed consent prior to participating in the study, in addition to specific consent for genetic analysis. Anonymity of participants remains ensured by providing only coded electronic data containing no information that can be used to identify individuals. Ethical approval for the research was obtained from the Health Research Ethics Committee of the North-West University (04M10 and NWU-00058-16-S1) and the study was conducted in accordance with the revised version (2000) of the Helsinki Declaration of 1975.

### Blood collection and storage

Fasting blood samples were collected between 7:00 and 11:00 am, and centrifuged within 30 minutes of collection at 2000 x *g* for 15 minutes. Blood samples for haemostatic variables were collected in 3.2% sodium citrate tubes, for lipid and IL-6 analyses in tubes without anti-coagulants and for glycated haemoglobin (HbA1_c_) in sodium fluoride tubes. Following centrifugation, samples were snap-frozen and stored at –80°C until analysis.

### Biochemical analyses

HbA1_c_ concentrations were quantified using a hexokinase method from Synchron^®^ systems (Beckman Coulter Co., Fulleron, CA, USA), and the D-10 haemoglobin testing system (Bio-Rad, Hercules, California, USA). A Sequential Multiple Analyser Computer, using the Konelab™ auto-analyser (Konelab 20i, Thermo Fischer Scientific, Vantaa, Finland) was used to measure high-density lipoprotein cholesterol (HDL-c) concentrations. Plasminogen activator inhibitor type-1 activity (PAI-1_act_) was quantified by an indirect enzymatic method (Spectrolyse PAI-1, Trinity Biotech, Bray, Ireland). Serum IL-6 was measured by means of the Elecsys, through ultra-sensitive enzyme immunoassays (Elecsys 2010, Roche, Basel, Switzerland).

Total fibrinogen concentrations were quantified using the modified Clauss method on the Dade Behring BCS coagulation analyser (Multifibrin U-test Dade Behring, Deerfield, IL, USA). An enzyme-linked immunosorbent assay using a 2.G2.H9 mouse monoclonal coating antibody against human γ’ fibrinogen (Santa Cruz Biotechnology, Santa Cruz, USA) and a goat polyclonal horseradish peroxidase-conjugated antibody against human fibrinogen (Abcam Cambridge, USA) was used to measure γ’ fibrinogen concentrations. Fibrinogen γ’ is expressed both as absolute concentration and as a percentage of total fibrinogen (%γ’).

Plasma fibrinolytic potential was determined by measuring turbidity with a spectrophotometer (A_405_) [42]. Tissue plasminogen activator (tPA; Actilyse, Boehringer Ingelheim, Ingelheim, Germany) was added to plasma clots induced by tissue factor (TF; Dade Innovin, Siemens Healthcare Diagnostics Inc., Marburg, Germany) according to the method of Lisman *et al*. [43]. The tPA and TF concentrations were modified to obtain a clot lysis time (CLT) between 60 and 100 minutes in the external control sample (pooled plasma) included in each run. Final concentrations in the plasma clots were 125 x diluted TF (approximately 59 pmol/L), 100 ng/mL tPA, 17 mmol/L CaCl_2_, and 10 mmol/L phospholipid vesicles (Rossix, Mölndal, Sweden). Resultant turbidity curves were analysed using Origin^®^ software version 8.5 (Origin lab^®^, 2010). CLT (minutes) was calculated as the difference between the time at the midpoint of clear and maximum turbidity (clot formation) and the midpoint between maximum and clear turbidity (clot lysis). In addition, lag time (minutes) was calculated as an indicator of the time required for the activation of the coagulation cascade by TF and for protofibrils to reach sufficient length to allow lateral aggregation. The slope of the curve (x10^-3^ au/s) during clot formation was used as a representation of the rate of lateral aggregation of the fibrin protofibrils. Lastly, maximum absorbance, the increase in absorbance at the peak of the curve (nm), was used as an indicator of fibre diameter.

### DNA isolation, SNP selection and genotyping

Genomic DNA was extracted from buffy coat using the QIAGEN FlexiGene™ kit. The quality of the DNA was determined using the NanoDrop™ spectrophotometer (ND-1000, Wilmington, DE, USA). Fourteen SNPs, spanning the three fibrinogen genes, were selected for genotyping based on literature identifying them as the top SNPs to demonstrate independent and IL-6-induced associations with total or γ’ fibrinogen concentrations [9, 11, 13, 44–49]. Furthermore, the promoter region of *FGB* was sequenced in a subgroup of 28 randomly selected individuals for the identification of novel SNPs. ABI Prism^®^, BIGDye^®^ Terminator version 3.1 Ready Reaction Cycle Sequencing Kits (Applied Biosystems, CityFoster, CA, USA) were used, and electropherograms were aligned using BioEdit (version 7.1.3.0, Ibis Biosciences, Carlsbad, CA, USA).

The polymorphisms selected for genotyping were *FGB*-rs7439150, rs2227385 (novel), rs1800789 (-1420_*G/A*_), rs2227388 (novel), rs1800791 (–854_*G/A*_), rs1800790 (–455_*G/A*_), rs1800788 (-249_*C/T*_), rs1800787 (–148_*C/T*_), rs4220, rs4463047, *FGA*-rs6050, rs2070011 (2224_*G/A*_), and *FGG*-rs2066865 and rs1049636 (9340_*T/C*_). Three methods were used to genotype these polymorphisms, of which two (Thermo Fischer Scientific^®^ Taqman based assays and the Illumina^®^ VeraCode GoldenGate assay technology using a BeadXpress^®^ platform), have been described previously [39]. In addition, *FGB*-rs1800789, rs1800790, rs4463047 and rs7439150 were genotyped by competitive allele-specific polymerase chain reactions (KASP) with supplies obtained from LGC Limited. Custom-designed assays and synthetic controls were manufactured by KBioscience and are presented in S1 Table (LGC, Middlesex, TW11 0LY, UK).

A two-step 61–to–55°C touchdown polymerase chain reaction (PCR) protocol was performed in a Hydrocycler 4^TM^ water bath thermal cycler (LGC, Middlesex, TW11 0LY, UK). The fluorescent signal of the PCR products was measured by a FLUOstar Omega SNP plate reader (BMG LABTECH Ltd) and the data were analysed by means of KlusterCaller™ V. 3.4.1.36 software (LGC, Middlesex, TW11 0LY, UK).

### Statistical analyses

The statistical analyses were performed in three phases. Firstly, the SNPs were investigated in terms of their location, LD and haplotypes using the Ensembl database release 84 [50] and Haploview version 4.2 [51]. In addition, their independent associations with phenotype outcomes (total and γ’ fibrinogen concentration, lag time, slope, maximum absorbance and CLT) were determined by independent t-tests and analysis of co-variance (ANCOVA) adjusting for age, gender, body mass index (BMI), human immunodeficiency virus (HIV) status, HbA1c and HDL-c. When CLT was used as an outcome variable, it was also adjusted for PAI-1_act_.

The second phase involved investigating the association of the fibrinogen phenotypes with IL-6 independent from the polymorphisms. Differences in phenotypes were tested with analysis of variance (ANOVA) and ANCOVA using IL-6 stratified by quartiles as the categorical variable. Tukey’s honest significant difference *post-hoc* tests were performed to determine significant inter-group differences.

Lastly, interaction effects between IL-6 and the fibrinogen polymorphisms on the fibrinogen phenotypes were determined by creating interaction terms and entering them into an ANCOVA with full factorial analysis. Adjustments were made for the same covariates listed above. Multiple testing was accounted for by performing Benjamini and Hochberg adjustments. Q-Q plots were used to evaluate the normality of the standardised residuals. Interaction data are reported as the slope of the linear regression line between IL-6 and the fibrinogen phenotypes split according to genotype.

In order to determine the possibility of a cumulative effect of the SNPs on the IL6-fibrinogen relationship, polymorphisms indicating significant interactions were grouped by means of the generation of a simple genetic ‘risk score’. Each genotype forming part of the model (significant interaction upon adjustment for multiple testing), was given a score per individual genotype. A score of zero was given for major allele homozygotes; one was given to the heterozygotes and two to minor allele homozygotes. Where the minor allele frequency (MAF) was low and only two groups were compared (see below), a score of one was given to all minor allele carriers.

Based on the minor allele frequency (determined by 1 out of 28 individuals showing variation at any given SNP in the *FGB* promoter region, upon sequencing) the power calculation indicated that in order to obtain 80% power to detect a medium effect size (Cohen’s d-value of 0.3), the genotype groups should contain a minimum of 71 individuals [52]. Analyses for SNPs having fewer than 71 individuals in the minor allele homozygote group were, therefore, performed using two groups only, combining heterozygotes and minor allele homozygotes.

Any analyses (t-tests, ANOVA, full factorial analysis ANCOVA) yielding significant results in terms of total or γ’ fibrinogen were followed by adjustments for these proteins during statistical testing using clot properties as outcome variables. These adjustments were made to identify SNP/IL-6-clot property outcomes that were not mediated by total and/or γ’ fibrinogen concentration. The statistical package for the social sciences (SPSS^®^) version 23 (IBM^®^ Corp, 2015) and Statistica^®^ version 13 (Statsoft Inc., Tulsa, OK, USA) were used for the above described analyses.

## Results

### Description of individual polymorphisms

**Fig 1:**
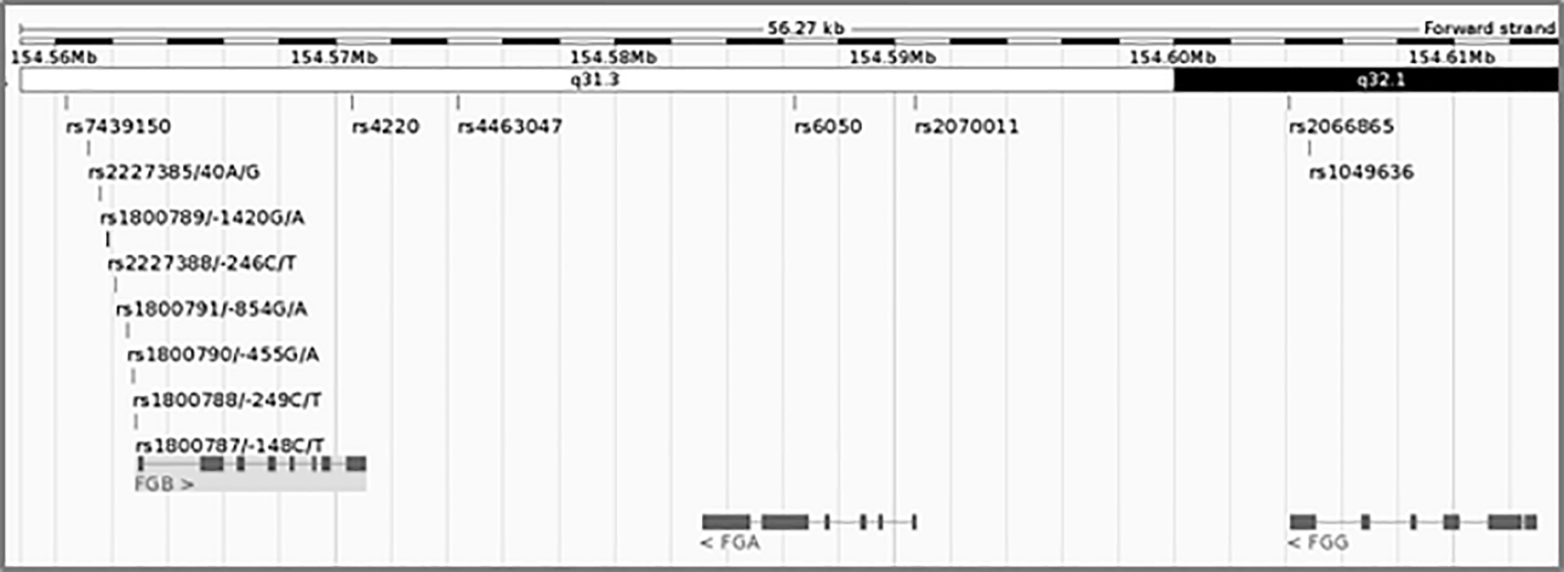
Fourteen polymorphisms spanning the fibrinogen gene cluster. Image generated through the Ensembl database [50].

Ten of the 14 investigated polymorphisms are situated in and around the β chain gene, of which eight are within the promoter area, one in exon 8 (Arg478Lys, previously reported as Arg448Lys) and one in the 3’ untranslated region (UTR). Two α chain variants, one in exon 2 (Thr331Ala, previously reported as Thr312Ala) and one in the promoter area, and two γ chain variants, both in the 3’ UTR, are also included. The relative positions of these SNPs, spanning a 50 Kb region on chromosome 4, are illustrated in Fig 1. All the variants were in Hardy-Weinberg equilibrium.

### LD and haplotype construction

**Fig 2:**
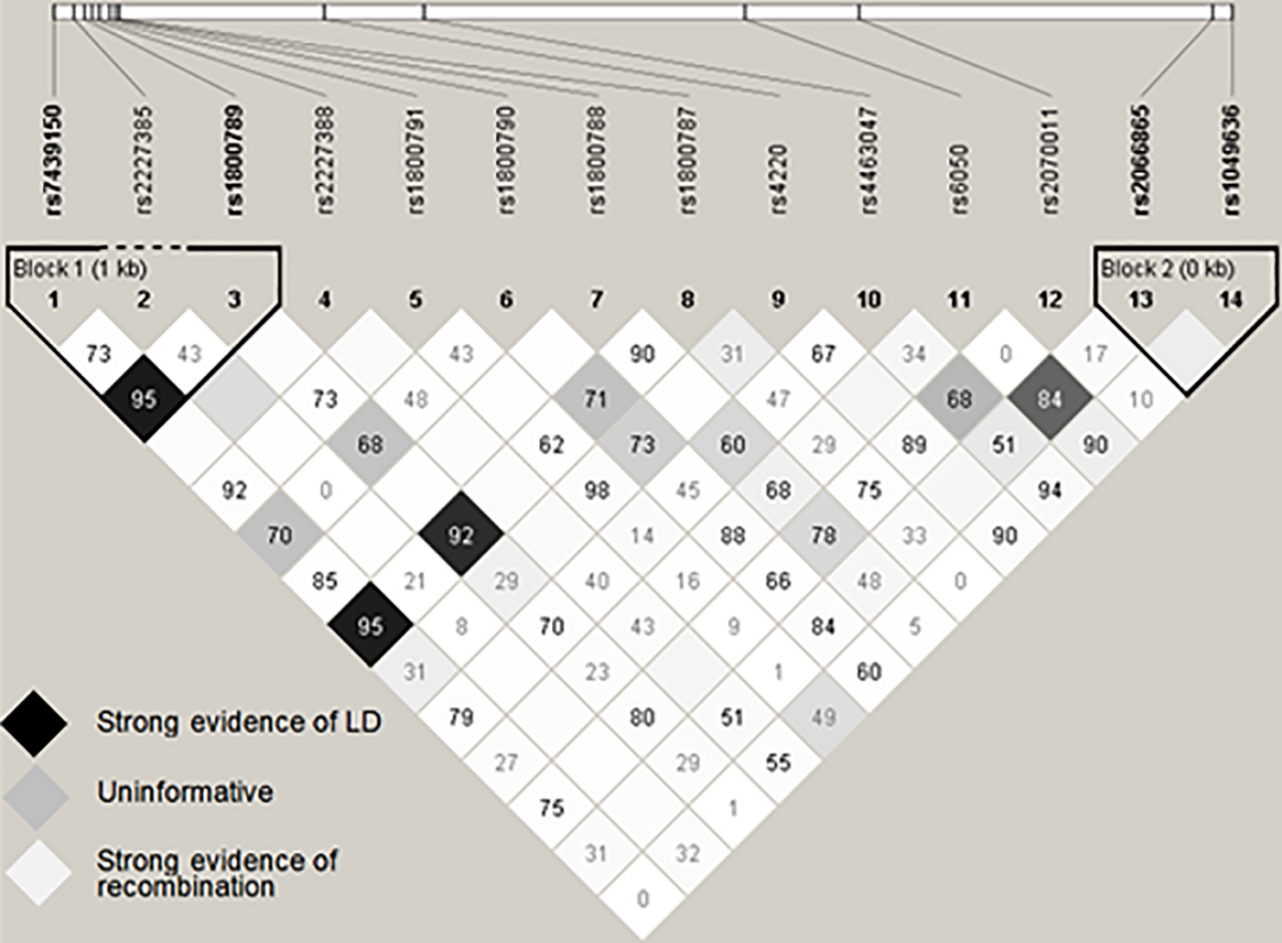
Pairwise LD structure of 14 fibrinogen SNPs, illustrated by D’ values on an r^2^ colour scheme. Empty boxes indicate D’ values of 1.0. Increased numerical values indicate stronger evidence of LD. Image generated through Haploview software [51].

Fig 2 depicts the LD pattern (illustrated by D’ values upon an r^2^ colour scheme) observed for the 14 SNPs. Lower recombination rates are indicated by increased numerical values (D’) and darker coloured blocks (r^2^). Blocks containing no numerical value indicate a D’ of 1.0. Two haplotype blocks *FGB*-rs7439150*rs2227385*rs1800789 and *FGG* rs2066865*rs1049636 were constructed *via* the method suggested by Gabriel *et al*. [53]. No complete LD (D’ and r^2^ = 1.0) was observed, as the LD pattern was disturbed owing to differing MAFs that led to relatively low r^2^ values. Acknowledging the limited LD, all further analyses were performed using individual polymorphisms only.

### Associations of individual polymorphisms with fibrinogen-related phenotypes

Population characteristics and associations of ten of the 14 SNPs with fibrinogen-related phenotypes have been reported previously [39] and are shown in the online supporting information only (S2, S3 and S4 Tables). Briefly, *FGB* –854_*A*_ and *FGG*-rs1049636_*C*_ were positively associated with total fibrinogen (p = 0.04 and 0.0009), and *FGA*-rs2070011_*A*_ with higher γ’ fibrinogen concentrations (p = 0.008). In terms of clot properties, *FGB* –148_*T*_ was associated with a larger fibre diameter (p = 0.001), whereas *FGA*-rs2070011_*A*_ and *FGG*-rs1049636_*C*_, lost significance in terms of their effect on maximum absorbance (fibre diameter) upon adjustment for γ’ and total fibrinogen, respectively (p = 0.06 and 0.33).

Associations of each of the four newly investigated promoter region SNPs with the outcome phenotypes are shown in Table 1. *FGB* –1420_*A*_ was significantly associated with total fibrinogen concentrations (p = 0.02) and the same trend was observed for *FGB*-rs7439150_*A*_ and *FGB* –455_*A*_ (p ≈ 0.05). Larger fibre diameter and longer CLT were observed in the presence of the *FGB* –1420_*A*_ allele (p = 0.04 and 0.02). In addition, *FGB* –455_*A*_ and *FGB*-rs7439150_*A*_ were positively associated with γ’ fibrinogen concentrations (p = 0.02 and 0.005), and *FGB*-rs7439150_*A*_ with fibre diameter (p = 0.02). Associations with clot properties were largely mediated by the primary fibrinogen associations, as most of the significance was lost upon adjustment for total and/or γ’ fibrinogen, apart from the association between *FGB* –1420_*A*_ and CLT, which remained (p = 0.03).

**Table 1:** Association of selected upstream *FGB* polymorphisms with fibrinogen-related phenotypes.

	Genotype	*FGB*-rs7439150^δ^	*FGB*-rs1800789^δ^	*FGB*-rs1800790	*FGB*-rs4463047
SNP pseudonym			*FGB*-1420 *G*>*A*	*FGB*-455 *G*>*A*	
Minor allele frequency (%)		A = 6.95	A = 6.78	A = 3.32	T = 11.4
Genotype group size (n)	major allele hz minor allele carrier	GG^1^ (1548) GA/AA^2^ (215)	GG^1^ (1513) GA/AA^2^ (292)	GG^1^ (1652) GA/AA^2^ (108)	CC^1^ (1424) CT/TT^2^ (357)
**Total fibrinogen** (g/L)	major allele hz	3.66 ± 2.13	3.64 ± 2.12 *	3.66 ± 2.12	3.71 ± 2.18
	minor allele carrier	3.97 ± 2.29	3.96 ± 2.29 *	4.05 ± 2.34	3.52 ± 1.99
**Fibrinogen γ**' (mg/mL)	major allele hz	0.37 ± 0.25*	0.38 ± 0.25	0.38 ± 0.26 *	0.38 ± 0.27
	minor allele carrier	0.41 ± 0.32*	0.42 ± 0.33	0.46 ± 0.37 *	0.37 ± 0.22
**Fibrinogen γ'** (%)	major allele hz	12.1 ± 8.07	12.3 ± 8.27	12.1 ± 8.01	12.1 ± 8.27
	minor allele carrier	11.6 ± 8.82	11.6 ± 7.80	13.2 ± 10.1	12.2 ± 7.90
**Lag time** (min)	major allele hz	6.47 ± 1.97	6.47 ± 1.96	6.48 ± 1.99	6.47 ± 1.97
	minor allele carrier	6.59 ± 1.96	6.55 ± 1.99	6.49 ± 1.98	6.52 ± 1.98
**Slope** (x10^−3^ au/s)	major allele hz	9.58 ± 4.42	9.54 ± 4.35	9.65 ± 4.36	9.67 ± 4.41
	minor allele carrier	10.0 ± 3.99	10.3 ± 4.09	9.75 ± 3.95	9.60 ± 4.32
**Maximum absorbance** (nm)	major allele hz	0.43 ± 1.59 *	0.43 ± 0.16 *	0.43 ± 0.16	0.43 ± 0.16
	minor allele carrier	0.46 ± 0.17 *	0.46 ± 0.17 *	0.46 ± 0.19	0.44 ± 0.16
**Clot lysis time** (min)	major allele hz	56.9 ± 11.2	56.8 ± 11.2 *^#^	57.0 ± 11.3	57.3 ± 11.3
	minor allele carrier	58.0 ± 12.0	58.3 ± 12.1 *^#^	58.7 ± 11.1	56.3 ± 11.3

A = adenine; C = cytosine; FGB = fibrinogen beta chain gene; G = guanine; rs = reference sequence; T = thymine; Lag time = time required for the activation of the coagulation cascade by TF and for protofibrils to reach sufficient length to allow lateral aggregation; Slope = rate of lateral aggregation of fibrin protofibrils; Maximum absorbance = indicator of fibre diameter.

Data presented as mean ± SD.

*p < 0.05

^#^ p < 0.05 upon adjustment for total and γ’ fibrinogen

^δ^ Strong evidence of LD (r^2^ = 0.89; D’ = 0.95).

Age, gender, BMI, HIV-status, HbA1_c_ and HDL-c were covariates. Associations with CLT were adjusted for PA-I1_act_ additionally.

### The association of IL-6 with fibrinogen-related phenotypes

**Fig 3:**
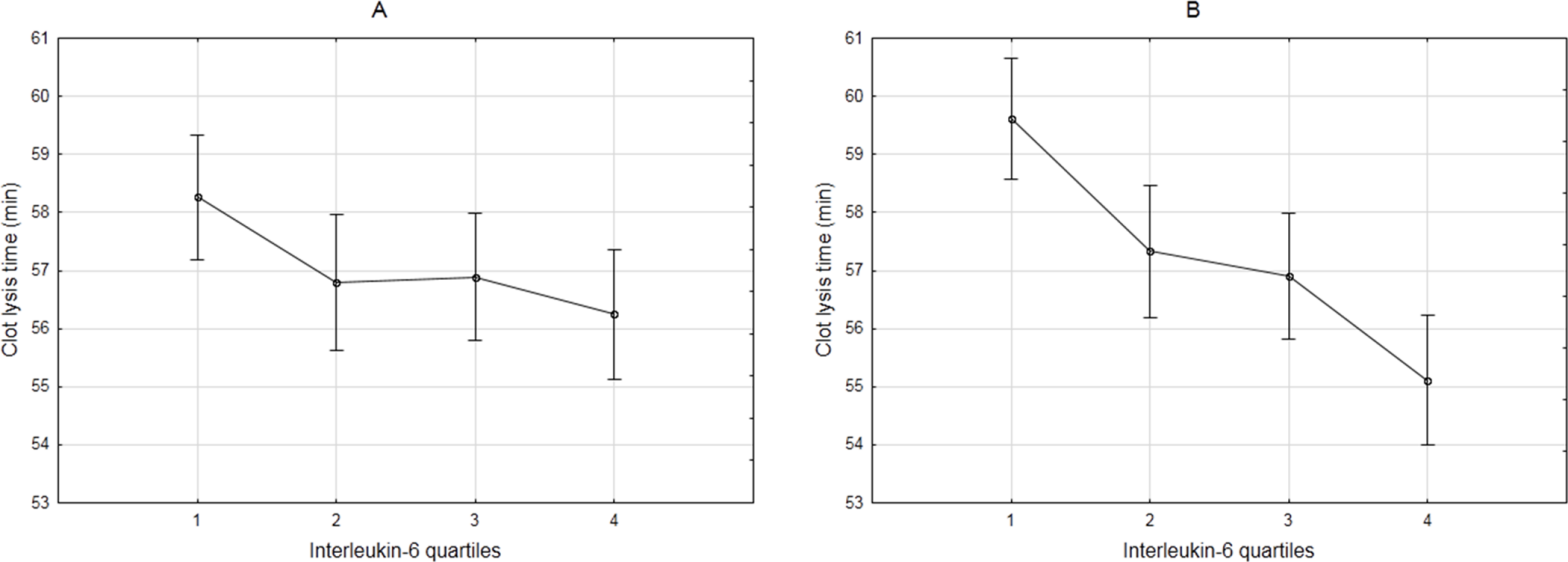
**CLT across IL-6 quartiles before (A) and after (B) adjustment for total fibrinogen, γ’ fibrinogen and PAI-1_act_.** Vertical bars denote 95% confidence interval. p-values: 0.066 (A); <0.00001 (B). Images are equally scaled.

The basic descriptive characteristics of the study population have been reported in a previous publication [39]. Descriptive statistics for fibrinogen and clot properties, including their association with IL-6 (presented as quartiles), are shown in Table 2. The mean IL-6 concentration was 6.50 ± 21.0 pg/mL. Total and γ’ fibrinogen concentrations were positively, although not linearly, associated with IL-6 with significantly higher concentrations in the fourth IL-6 quartile compared to the first three quartiles. The % γ’ fibrinogen did not reach significance, implying that the fibrinogen γ’ association is probably a reflection of the association of IL-6 with total fibrinogen. Positive associations were also observed for lag time, slope and maximum absorbance, although subsequent adjustment for total and γ’ fibrinogen led to loss of significance. CLT decreased as IL-6 increased, with total fibrinogen concentration attenuating this effect. Adjustments for total and γ’ fibrinogen, and thereafter PAI-1_act_ (as a main modulator of CLT) significantly increased this negative association (Fig 3).

**Table 2:** Outcome phenotypes descriptive statistics and association with IL-6- quartiles.

Variable	Whole group (n)	Interleukin-6 quartiles (pg/mL)				p-value unadjusted (adjusted)
		Quartile 1 > 0.76	Quartile 2 1.50–2.84	Quartile 3 2.85–5.75	Quartile 4 5.76–424	
**Total fibrinogen** (g/L)	3.69 ± 2.18 (1593)	3.02 ± 1.62 ^a^^b^	3.32 ± 1.86 ^c^^d^	3.95 ± 2.17 ^a^^c^^e^	4.53 ± 2.66 ^b^^d^^e^	<0.001
**Fibrinogen γ'** (mg/L)	0.38 ± 0.27 (1591)	0.32 ± 0.22 ^a^^b^	0.36 ± 0.25 ^c^	0.39 ± 0.21 ^a^^d^	0.46 ± 0.37 ^b^^c^^d^	<0.001
**Fibrinogen γ'** (%)	12.1 ± 8.25 (1527)	12.5 ± 8.56	12.4 ± 7.58	11.8 ± 7.77	11.8 ± 8.94	0.552
**Lag time** (min)	6.46 ± 1.97 (1480)	6.24 ± 1.99 ^a^	6.46 ± 2.03	6.64 ± 1.93 ^a^	6.38 ± 1.94	0.033 (0.126)
**Slope** (x10^−3^ au/s)	9.70 ± 4.42 (1492)	9.19 ± 3.80 ^a^	9.60 ± 4.37	9.86 ± 4.30	10.5 ± 5.15 ^a^	<0.001 (0.897)
**Maximum absorbance** (nm)	0.43 ± 0.16 (1445)	0.41 ± 0.14 ^a^^b^	0.43 ± 0.15	0.45 ± 0.15 ^a^	0.45 ± 0.18 ^b^	<0.001 (0.183)
**CLT** (min)	57.3 ± 11.2 (1591)	58.3 ± 10.8 ^1^	56.9 ± 10.9	56.8 ± 11.5	56.2 ± 11.9 ^1^	0.066 (0.002)

Data presented as mean ± SD

Adjusted p-value = adjusted for total and γ’ fibrinogen; CLT = clot lysis time; Lag time = time required for the activation of the coagulation cascade by TF and for protofibrils to reach sufficient length to allow lateral aggregation; Slope = rate of lateral aggregation of fibrin protofibrils; Maximum absorbance = indicator of fibre diameter.

^abcde^ Means with the same symbol differ significantly for individual outcome variables

^1^ Means with the same numerical value differ significantly for individual outcome variables upon adjustment.

### Genotype-IL-6 interactions in terms of phenotype predictions

Interaction analyses were performed for each polymorphism with IL-6 in relation to each of the fibrinogen measures and clot properties. The association between IL-6 and fibrinogen phenotype, stratified according to genotype for each of the significant polymorphisms is reported in Tables 3 and 4. IL-6 concentration was associated with a steeper increase in fibrinogen concentration in minor allele carriers of seven SNPs and a shallower increase in total fibrinogen in *FGA*-rs6050_*G*_. For *FGG*-rs2066865, a shallower increase was observed in the presence of one minor allele, with a steeper increase in the presence of two minor alleles, more so than for the common allele homozygotes. Upon adjusting for multiple testing, IL-6 did not significantly interact with any of the investigated SNPs to influence fibrinogen γ’. IL-6 interacted with six SNP’s in determining fibre diameter (maximum absorbance), in which the presence of the minor allele resulted in a stronger positive association between IL-6 and maximum absorbance than the respective major alleles. Adjustment for fibrinogen concentration largely nullified these associations, with only two associations remaining significant (*FGB*-rs7439150 and –148_*C/T*_). *FGB–*1420_*G/A*_, which is in high LD with *FGB*-rs7439150 and –148_*C/T*_, however, lost its significance after adjustment for fibrinogen owing to its independent association with fibrinogen concentration. No significant interactions were observed for lag time, slope or CLT. Removal of individuals with IL-6 concentrations higher than 100 pg/ml (n = 8) resulted in a loss of significance for many of the interactions presented in Tables 3 and 4. The only remaining IL-6 interaction was with *FGG-*rs1049636 in terms of fibrinogen concentration [p < 0.001, slope 0.056 (0.041–0.071) for the major allele carriers and slope 0.110 (0.082–0.138) for the minor allele carriers].

**Table 3:** Genotype-IL-6 interactions modulating total fibrinogen concentrations.

Phenotype	Gene	IL-6 interaction with SNP	Interaction p-value			Major allele homozygotes		Heterozygotes or minor allele carriers (where only two groups)		Minor allele homozygotes (where three groups)
					n	Slope* (95% CI)	n	Slope* (95% CI)	n	Slope* (95% CI)
**Total fibrinogen** (g/L)	*FGB*	rs7439150^δ^	0.001	GG	1293	0.015 (0.009–0.020)	174	0.072 (0.040–0.104)		
	*FGB*	rs1800789^δ^	<0.001	GG	1263	0.012 (0.006–0.018)	240	0.071 (0.050–0.093)		
	*FGB*	rs1800788	<0.001	CC	1419	0.017 (0.012–0.023)	154	0.083 (0.045–0.121)		
	*FGB*	rs1800787^δ^	0.001	CC	1164	0.016 (0.010–0.022)	152	0.077 (0.043–0.111)		
	*FGB*	rs4220	0.003	GG	1121	0.016 (0.010–0.022)	224	0.061 (0.035–0.087)		
	*FGA*	rs6050	<0.001	AA	676	0.039 (0.028–0.049)	526	0.016 (0.006–0.027)	132	1 x 10^−4^(-0.010–0.010)
	*FGA*	rs2070011	0.010	GG	925	0.015 (0.009–0.021)	416	0.042 (0.024–0.060)		
	*FGG*	rs2066865	<0.001	CC	746	0.040 (0.029–0.050)	482	0.005 (-0.003–0.012)	108	0.111 (0.067–0.156)
	*FGG*	rs1049636	<0.001	TT	975	0.014 (0.008–0.020)	371	0.110 (0.082–0.138)		

A = adenine; C = cytosine; CI = confidence interval; G = guanine; IL-6 = interleukin-6; rs = reference sequence; T = thymine; rs1800789 = -1420_*G/A*_; rs1800788 = -249_*C/T*_; rs1800787 = -148_*C/T*_

rs1800790 = -455_*G/A*_; rs2070011 = 2224_*G/A*_, rs1049636 = 9340_T/C_

^δ^Variants in high linkage disequilibrium (r^2^ > 0.82; D’ > 0.92)

*In the regression line y = **m**x + c, “slope” refers to **m**.

**Table 4:** Genotype-IL-6 interactions modulating clot properties.

Phenotype	Gene	IL-6 interaction with SNP	Interaction p-value				Major allele homozygotes		Heterozygotes or minor allele carriers (where only two groups)		Minor allele homozygotes (where three groups)
			Unadjusted	Adjusted							
						n	Slope* (95% CI)	n	Slope* (95% CI)	n	Slope* (95% CI)
**Maximum absorbance** (nm)	*FGB*	rs7439150 ^δ^	<0.001	0.01	GG	1274	1x10^-4^ (-3x10^-4^–0.001)	179	0.003 (0.002–0.003)		
	*FGB*	rs1800789 ^δ^	<0.001	0.82	GG	1245	2x10^-4^ (-3x10^-4^–0.001)	241	0.002 (0.002–0.003)		
	*FGB*	rs1800788	0.001	0.04	CC	1407	0.001 (2x10^-4^–0.001)	153	0.004 (0.002–0.007)		
	*FGB*	rs1800787 ^δ^	<0.001	0.01	CC	1157	1x10^-4^ (-3x10^-4^–0.001)	157	0.003 (0.002–0.003)		
	*FGA*	rs6050	0.001	0.04	AA	663	-1x10^-5^ (-0.001–0.001)	531	0.002 (0.001–0.002)	133	1x10^-5^(-0.001–0.001)
	*FGG*	rs1049636	0.003	0.23	TT	970	0.001 (3x10^-4^–0.001)	369	0.003 (0.001–0.005)		

A = adenine; C = cytosine; CI = confidence interval; G = guanine; IL-6 = interleukin-6; rs = reference sequence; T = thymine; rs1800789 = -1420_*G/A*_; rs1800788 = -249_*C/T*_; rs1800787 = -148_*C/T*_; rs1049636 = 9340_*T/C*_

Adjusted = adjusted for total fibrinogen (g/L); Slope = rate of lateral aggregation of fibrin protofibrils; Maximum absorbance = indicator of fibre diameter.

^δ^Variants in high linkage disequilibrium (r^2^ > 0.82; D’ > 0.92)

*In the regression line y = **m**x + c, “slope” refers to **m**.

**Fig 4:**
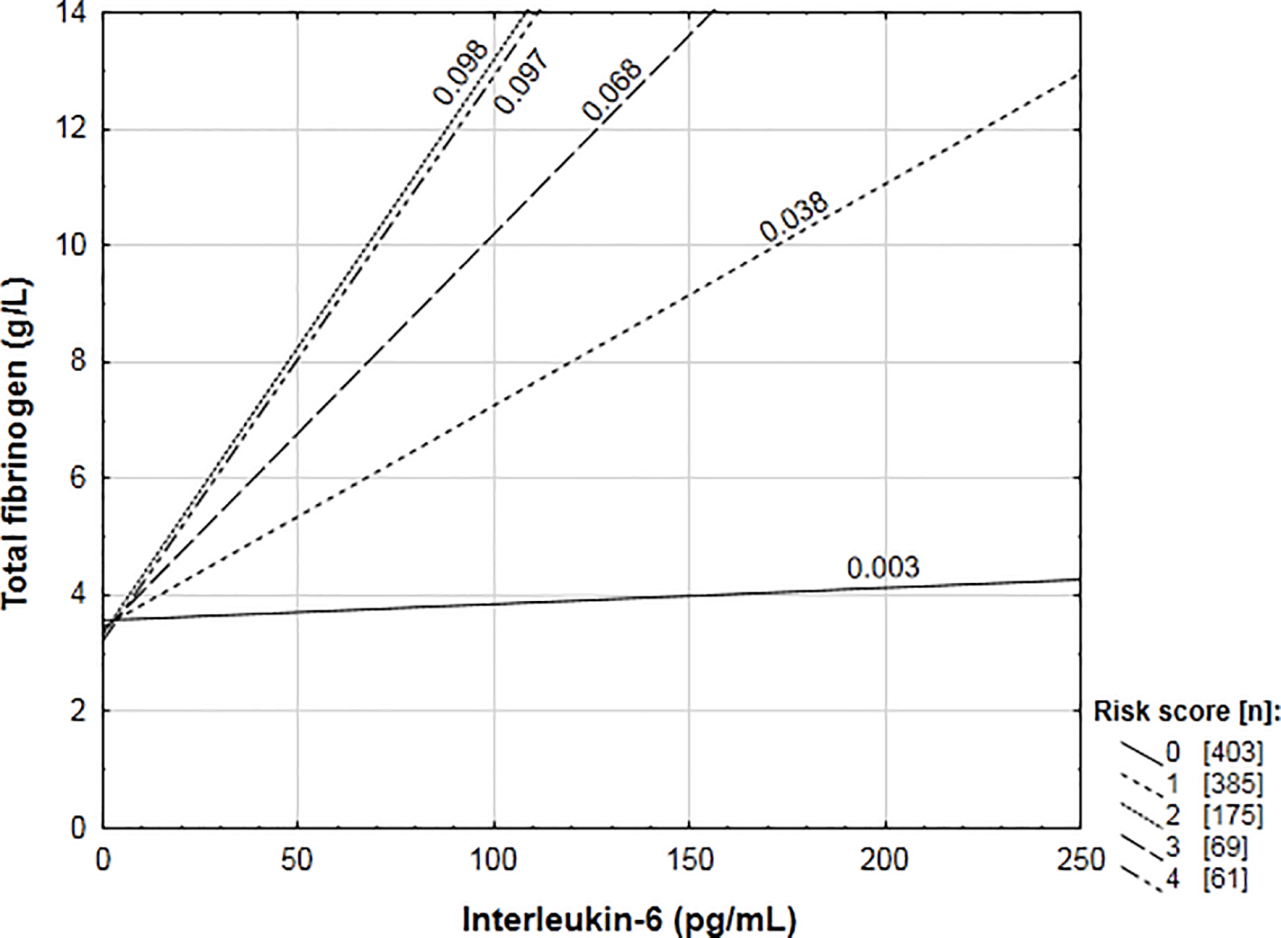
Association of total fibrinogen concentrations with circulating interleukin-6 by risk score groups.

In addition to the individual interaction analyses, the seven variants resulting in a steeper fibrinogen increase in the presence of IL-6 (Table 3) were grouped in a genetic ‘risk score’ to determine whether there were additive effects when these genotypes occurred together in an individual. Each individual’s score was composed of the sum of the seven polymorphisms allocated a value of either zero (major allele homozygote) or one (minor allele carrier). Final scores ranked from zero to six, therefore, scores represented the presence of zero to six minor allele groups. The risk score showed a significant interaction with IL-6 (p < 0.001) in the prediction of fibrinogen concentrations. Fig 4 schematically represents the slope the association between IL-6 and fibrinogen for each score. The addition of a minor allele group to the risk score increased the slope of the IL-6-fibrinogen association. The fifth and sixth risk score were removed from Fig 4 as the sizes of these groups were too small to provide adequate power (n = 24 and 5, respectively). The interaction, however, remained significant upon removal of these groups (p < 0.001). The regression coefficient for the line indicating the IL-6–fibrinogen association for risk score one differed significantly from risk score zero (p = 0.04), with two, three and four differing from one (p = 0.01, 0.048, 0.005, respectively) but not from each other, indicating a possible threshold to the additive effect when more than three risk alleles occurred together.

## Discussion

High LD in the fibrinogen gene cluster in Europeans continues to hinder the identification of functional polymorphisms contributing to fibrinogen concentration and functionality (specifically fibrin clot properties). This becomes even more complex when taking the role of transcriptional enhancers, such as IL-6, into consideration. Although little is known about the fibrinogen genes in Africans, preliminary evidence suggests distinct genetic differences from that of Europeans, including higher recombination rates and polymorphic variance. In addition, this population is known for altered fibrinogen and IL-6 phenotypes. This study, therefore, aimed to identify possible functional fibrinogen SNPs and novel IL-6-interactions related to total and γ’ fibrinogen, as well as indicators of clot formation, structure and lysis by using the unique African genetic and phenotypic profile. Our data revealed that none of the common European fibrinogen haplotypes is present in this African population. We demonstrate, in a population with chronic low-grade inflammation, that IL-6 interacted with several of the fibrinogen SNPs to influence fibrinogen concentration and even resulted in altered fibrin clot properties. A novel finding was that IL-6 not only interacted with individual SNPs, but that an additive effect was also observed when harbouring more than one risk allele concurrently.

The MAF of 12 of the 14 investigated SNPs was significantly lower in this Tswana population than that reported globally [50]. No variation has been reported for the *FGB*-rs2227385 and *FGB*-rs2227388 SNPs outside Africa [50]. As predicted, a high recombination rate was observed in this study population, resulting in no complete LD among any of the SNPs. However, strong LD between *FGB* -1420_*G/A*_ and -148_*C/T*_ was observed, similar to the finding of Dehghan *et al*. [54], although the LD observed for *FGB*-rs7439150 with these SNPs is novel. This lack of full LD differs from numerous reports of almost complete LD between the –1420_*G/A*_, –933_*C/T*_, –455_*G/A*_ and –148_*C/T*_ SNPs in Europeans [11, 16, 17, 49, 55–57]. None of the frequently occurring haplotypes, consisting of –1420_*G/A*_, –933_*C/T*_, –854_*G/A*_, –455_*G/A*_, –249_*C/T*_ and –148_*C/T*_, [11, 16, 17], was present in this study population, highlighting the genetic diversity in Africans. In addition, *FGB*-933_*C/T*_ revealed no genetic variation in this population.

In the present study, *FGB –*854_*A*_, –1420_*A*_ and *FGG*-rs1049636_*C*_ were associated with higher total fibrinogen concentrations. Higher γ’ fibrinogen concentrations (but not % γ’) were observed in the presence of *FGB* –455_*A*_, rs7439150_*A*_ and *FGA*-rs2070011_*A*_. Furthermore, a positive association between *FGB –*148_T_ and fibre diameter, and *FGB* –1420_*A*_ and CLT was observed. IL-6 correlated positively with total and γ’ fibrinogen, thereby accelerating clot formation and increasing fibre diameter. CLT was, however, negatively associated with IL-6, and both total and γ’ fibrinogen concentration attenuated this association. Five *FGB*, one *FGA* and one *FGG* SNP significantly interacted with circulating IL-6 to steepen the IL-6 fibrinogen association. These interactions proved to be additive, with the presence of more than one minor allele across the gene cluster resulting in greater increases in IL-6-induced fibrinogen expression, although a threshold was reached when more than three minor allele variants occurred together in an individual. Lastly, these interactive associations reflected functional effects in terms of the rate of lateral aggregation and fibre diameter, thus indicating a possible mechanism by which the fibrinogen SNPs, during the acute phase, could enhance thrombotic risk.

Individual SNP associations for ten of the polymorphisms investigated have been reported previously [39]. The associations of the four additionally genotyped SNPs, although not all reaching significance, are in agreement with the existing literature. Increased fibrinogen concentrations in the presence of *FGB*-rs7439150_*A*_, –1420_*A*_ and –455_*A*_ have been reported in case-control and genome-wide association analyses [9, 54, 56, 58–65]. *FGB*-rs4463047_T_ was negatively associated with total fibrinogen concentrations in three association studies [9, 61, 66], whereas we did not observe any association at this locus. The novel association of –1420_*A*_ with faster CLT, independent of fibrinogen concentration, deserves further investigation.

Most of the research into both the heritability of and variant associations with fibrinogen has been conducted in European populations. Although limited information is available for African-Americans [9, 66, 67], being an ad-mixed population, their genetic background also differs from individuals from continental Africans. The lack of LD and common haplotypes, alongside the inability to reproduce results of independent SNP associations in this current investigation, questions whether heritability estimates obtained in Europeans or African Americans can be extrapolated to Africans. Future studies should replicate the existing heritability studies in African ethnic sub-groups to address this. The 14 SNPs investigated here contributed a mere 0.5% to the variance in total fibrinogen.

Total fibrinogen and IL-6 concentrations were generally higher than those reported in Europeans [13, 59]. IL-6 correlated positively with fibrinogen concentration, thereby accelerating clot formation and increasing fibre diameter, consequently contributing to clot pathology associated with CVD [68]. In the present study, higher IL-6 was associated with a faster CLT, independent of fibrinogen concentration and clot structure. This is probably the result of increases in other biomarkers influencing CLT, such as plasminogen leading to faster clot lysis. Previous reports have positively associated IL-6 with increased plasminogen transcription [69, 70].

Fibrinogen γ’ was also positively associated with IL-6, although the non-significant association of IL-6 with the % γ’ fibrinogen revealed the observation to be largely a reflection of the relationship with total fibrinogen. This finding suggests that in this study population, characterised by chronic low-grade inflammation, the influence of IL-6 on total and γ’ fibrinogen is probably due to up-regulation of the entire fibrinogen gene cluster, while alterations in the *FGG* alternative splicing mechanism may be more relevant in pronounced inflammatory conditions such as have been reported for CVD [26, 27].

IL-6-induced fibrinogen gene expression can be altered by polymorphic variation [16, 17]. Therefore, it is important to take genotype-IL-6 interactions into consideration when determining the influence of genetic variance on the fibrinogen phenotype, particularly in populations with prevalent inflammatory conditions. Nine SNPs interacted significantly with IL-6 in predicting fibrinogen concentrations. These interactions furthermore led to altered fibrin clot properties, in particular increased fibre diameter, which has been associated with CVD [71]. The number of significant genotype-IL-6 interactions was reduced when individuals with high IL-6 concentrations (>100 pg/mL) were removed from analyses, suggesting that these interactions are physiologically more relevant in the presence of high IL-6 plasma concentrations. The interaction with *FGG*-rs1049636 remained, however, indicating its potential relevance to fibrinogen regulation during chronic low-grade inflammatory conditions.

In addition, these interactions had an additive effect that could only be detected because of the lack of LD in the fibrinogen gene cluster in this African study population. The additive effect reached a threshold when more than three risk alleles occurred together. This observation should be investigated in a larger study population to allow analysis of the number of risk alleles, rather than just the presence thereof, thereby exploring dominant/co-dominant/recessive effects. Nonetheless, the observed additive effects reflect true physiological mechanisms, as the particular combinations are harboured concurrently in each individual. This data are in agreement with the findings of Ken-Dror *et al*. [63] who suggested that the fibrinogen phenotype is not regulated by one functional SNP only, but by a combination of minor alleles spanning the whole cluster. The lack of a causal contribution of fibrinogen to CVD as suggested by Mendelian randomisation studies may, therefore, need to be revisited, because these studies investigated single variants only and focussed on fibrinogen concentration alone while omitting qualitative effects such as altered clot properties [72]. Although our sample size was relatively small for genetic association studies, it did allow for the detection of significant results. Future studies should include a large study population from genetically diverse ethnic groups for replication and validation of current results.

In this Tswana population, of the 14 SNPs investigated, *FGB*-rs7439150, –1420_*G/A*_ and –148_C/T_ had the most pronounced effects on the fibrinogen phenotype in terms of both concentration and altered clot properties, with several of these associations altered by IL-6 concentrations. Two of these SNPs, *FGB*-rs7439150 and –1420_G/A_ were also the top SNPs associated with fibrinogen concentrations in the largest fibrinogen GWAS to date (β = 0.031, p = 9.5E^-181^; β = 0.031, p = 6.9E^-180^, respectively) [61]. *FGB* –148_C/T_ was not investigated most likely due to complete linkage with *FGB* –1420_G/A_ in this European study population [61].

*FGB*-rs7439150 is located in a regulatory feature (ENSR00000175110) spanning 623 base pairs (4:154560017–154560640), and is predicted to have methylation potential, so could influence transcription [73]. In addition, possible functionality of –148_*C/T*_ has been described by Verschuur *et al*. [17] owing to its ability to alter fibrinogen’s response to IL-6 by interfering with the hepatocyte nuclear factor 3 and CCAAT enhancer binding protein binding sites. Although the association of the *FGB* promoter –1420_*G/A*_ polymorphism with fibrinogen concentration has been reported previously [9, 54, 61], it is not associated with any known regulatory features [73] and further investigation into its functionality is required. In this study population, these three SNPs were also in high, albeit not complete LD (D’ > 0.92, r^2^ > 0.82), therefore, the possibility of lack of independence cannot be excluded. They did, however, have unrelated independent genotype-phenotype associations; *FGB*-rs7439150 with fibrinogen γ, *FGB* –1420_*G/A*_ with total fibrinogen, and *FGB* –148_*C/T*_ with maximum absorbance, respectively. Distinct differences in their IL-6-interactive associations (Tables 3 and 4) were also observed, strengthening their hypothesised independent functional contribution to the fibrinogen-related phenotypes. The possibility that other SNPs in strong LD with those we genotyped, were responsible for the associations observed, can of course not be ruled out. In addition there are many more (novel and known) SNPs in the fibrinogen gene cluster, which were not investigated, that may also influence the fibrinogen phenotype [9, 61, 67].

## Conclusion

The genotypic exploration of the fibrinogen phenotype in the Tswana population showed the unique genetic composition of black South Africans even though this study did not attempt to characterise all the variation in the fibrinogen genes of this African population. It focused instead on SNPs previously indicated in the literature to have functional effects which could, to date, not be confirmed as a result of their high LD. It is clear that data obtained from European research cannot be extrapolated directly to Africans and that the risk profile in terms of the relative contribution of genetics and environmental factors might differ. Our results demonstrate that fibrinogen SNPs can modulate the influence of IL-6 on fibrinogen concentration individually and in an additive manner, with the presence of more minor alleles across these genes leading to greater increases in IL-6-induced fibrinogen expression in an apparently healthy African study population, albeit one with apparent chronic, low-grade inflammation. Therefore, in future, when investigating the effect of fibrinogen genetics on fibrinogen concentrations and CVD outcome, the possible interactions with modulating factors and the fact that SNP effects seem to be additive should be taken into account.

## Supporting information

S1 Table Primer and synthetic control sequences used for KASP analyses. (DOCX)

S2 Table Basic descriptive characteristics of the study population as published by Kotzé *et al*, (2015). (DOCX)

S3 Table Associations of individual SNPs with fibrinogen γ′, % γ′ and total fibrinogen as published by Kotzé *et al*, (2015). (DOCX)

S4 Table Associations of individual SNPs with clot-related phenotypes as published by Kotzé *et al*, (2015). (DOCX)

## References

[1] Sidelmann JJ, Gram J, Jespersen J, Kluft C. Fibrin clot formation and lysis: basic mechanisms. Semin Thromb Haemost; 2000;26: 605–618. 10.1055/s-2000-13216 11140797

[2] Davalos D, Akassoglou K. Fibrinogen as a key regulator of inflammation in disease. Semin Immunopathol; 2012;34: 43–62. doi: 10.1007/s00281-011-0290-8 22037947 10.1007/s00281-011-0290-8

[3] Danesh J, Lewington S, Thompson SG, Lowe GD, Collins R, Kostis JB, et al Plasma fibrinogen level and the risk of major cardiovascular diseases and nonvascular mortality: an individual participant meta-analysis. JAMA. 2005;294: 1799–1809. doi: 10.1001/jama.294.14.1799 16219884 10.1001/jama.294.14.1799

[4] Kaptoge S, Seshasai SRK, Gao P, Freitag DF, Butterworth AS, Borglykke A, et al Inflammatory cytokines and risk of coronary heart disease: new prospective study and updated meta-analysis. Eur Heart J. 2014;35: 578–589. doi: 10.1093/eurheartj/eht367 24026779 10.1093/eurheartj/eht367 PMC3938862

[5] Tzoulaki I, Murray GD, Lee AJ, Rumley A, Lowe GD, Fowkes FGR. Relative value of inflammatory, hemostatic, and rheological factors for incident myocardial infarction and stroke: the Edinburgh Artery Study. Circulation. 2007;115: 2119–2127. doi: 10.1161/CIRCULATIONAHA.106.635029 17404162 10.1161/CIRCULATIONAHA.106.635029

[6] Hinds DA, Buil A, Ziemek D, Martinez-Perez A, Malik R, Folkersen L, et al Genome-wide association analysis of self-reported events in 6135 individuals and 252 827 controls identifies 8 loci associated with thrombosis. Hum Mol Genet. 2016;25: 1867–1874. doi: 10.1093/hmg/ddw037 26908601 10.1093/hmg/ddw037 PMC4986325

[7] Keavney B, Danesh J, Parish S, Palmer A, Clark S, Youngman L, et al Fibrinogen and coronary heart disease: test of causality by ‘Mendelian randomization’. Int J Epidemiol. 2006;35: 935–943. doi: 10.1093/ije/dyl114 16870675 10.1093/ije/dyl114

[8] Meade TW, Humphries SE, De Stavola BL. Commentary: fibrinogen and coronary heart disease—test of causality by 'Mendelian' randomization by Keavney et al. Int J Epidemiol. 2006;35: 944–947. doi: 10.1093/ije/dyl149 16870676 10.1093/ije/dyl149

[9] Sabater-Lleal M, Huang J, Chasman D, Naitza S, Dehghan A, Johnson AD, et al Multiethnic meta-analysis of genome-wide association studies in >100 000 subjects identifies 23 fibrinogen-associated Loci but no strong evidence of a causal association between circulating fibrinogen and cardiovascular disease. Circulation. 2013;128: 1310–1324. doi: 10.1161/CIRCULATIONAHA.113.002251 23969696 10.1161/CIRCULATIONAHA.113.002251 PMC3842025

[10] de Lange M, Snieder H, Ariëns RAS, Spector TD, Grant PJ. The genetics of haemostasis: a twin study. Lancet. 2001;357: 101–105. doi: 10.1016/S0140-6736(00)03541-8 11197396 10.1016/S0140-6736(00)03541-8

[11] Green F. Fibrinogen polymorphisms and atherothrombotic disease. Ann N Y Acad Sci. 2001;936: 549–559. 11460514 10.1111/j.1749-6632.2001.tb03543.x

[12] Hamsten A, De Faire U, Iselius L, Blombäck M. Genetic and cultural inheritance of plasma fibrinogen concentration. Lancet. 1987;330: 988–991. 10.1016/s0140-6736(87)92557-8 2889959

[13] Jacquemin B, Antoniades C, Nyberg F, Plana E, Müller M, Greven S, et al Common genetic polymorphisms and haplotypes of fibrinogen alpha, beta, and gamma chains affect fibrinogen levels and the response to proinflammatory stimulation in myocardial infarction survivors: the AIRGENE study. J Am Coll Cardiol. 2008;52: 941–52. doi: 10.1016/j.jacc.2008.06.016 18772067 10.1016/j.jacc.2008.06.016

[14] Fish RJ, Neerman-Arbez M. Fibrinogen gene regulation. Thromb Haemost. 2012;108: 419–426. doi: 10.1160/TH12-04-0273 22836683 10.1160/TH12-04-0273

[15] Fuller GM, Zhang Z. Transcriptional control mechanism of fibrinogen gene expression. Ann N Y Acad Sci. 2001;936: 469–479. 11460505 10.1111/j.1749-6632.2001.tb03534.x

[16] Morozumi T, Sharma A, De Nardin E. The Functional Effects of the− 455G/A Polymorphism on the IL-6-Induced Expression of the β-fibrinogen Gene may be due to Linkage Disequilibrium with Other Functional Polymorphisms. Immunol Invest. 2009;38: 311–223. 19811441 10.1080/08820130902745153

[17] Verschuur M, de Jong M, Felida L, de Maat MP, Vos HL. A Hepatocyte Nuclear Factor-3 Site in the Fibrinogen β Promoter Is Important for Interleukin 6-induced Expression, and Its Activity Is Influenced by the Adjacent–148C/T Polymorphism. J Biol Chem. 2005;280: 16763–16771. doi: 10.1074/jbc.M501973200 15737987 10.1074/jbc.M501973200

[18] Brull D, Dhamrait S, Moulding R, Rumley A, Lowe G, Humphries S, et al The effect of fibrinogen genotype on fibrinogen levels after strenuous physical exercise. Thromb Haemost. 2002;87: 37–41. 11858186

[19] Cotton J, Webb K, Mathur A, Martin J, Humphries S. Impact of the-455G> A promoter polymorphism in the B fibrinogen gene on stimulated fibrinogen production following bypass surgery. Thromb Haemost. 2000;84: 926–927. 11127884

[20] Montgomery HE, Clarkson P, Nwose O, Mikailidis D, Jagroop I, Dollery C, et al The acute rise in plasma fibrinogen concentration with exercise is influenced by the G-453-A polymorphism of the β-fibrinogen gene. Arterioscler Thromb Vasc Biol. 1996;16: 386–391. 8630664 10.1161/01.atv.16.3.386

[21] Meh DA, Siebenlist KR, Mosesson MW. Identification and characterization of the thrombin binding sites on fibrin. J Biol Chem. 1996;271: 23121–23125. 8798504 10.1074/jbc.271.38.23121

[22] Rein-Smith CM, Anderson NW, Farrell DH. Differential regulation of fibrinogen γ chain splice isoforms by interleukin-6. Thromb Res. 2013;131: 89–93. doi: 10.1016/j.thromres.2012.09.017 23036532 10.1016/j.thromres.2012.09.017 PMC3530012

[23] Wolfenstein-Todel C, Mosesson MW. Human plasma fibrinogen heterogeneity: evidence for an extended carboxyl-terminal sequence in a normal gamma chain variant (gamma'). Proc Natl Acad Sci USA. 1980;77: 5069–5073. 6933547 10.1073/pnas.77.9.5069 PMC349997

[24] Duan HO, Simpson-Haidaris PJ. Functional Analysis of Interleukin 6 Response Elements (IL-6REs) on the Human γ-Fibrinogen Promoter. J Biol Chem. 2003;278: 41270–41281. doi: 10.1074/jbc.M304210200 12900415 10.1074/jbc.M304210200

[25] Zhang Z, Fuentes NL, Fuller GM. Characterization of the IL-6 responsive elements in the γ fibrinogen gene promoter. J Biol Chem. 1995;270: 24287–24291. 7592638 10.1074/jbc.270.41.24287

[26] Alexander KS, Madden TE, Farrell DH. Association between γ′ fibrinogen levels and inflammation. Thromb Haemost. 2011;105: 605–609. doi: 10.1160/TH10-09-0626 21174007 10.1160/TH10-09-0626 PMC4110682

[27] Cheung EY, Uitte de Willige S, Vos HL, Leebeek FW, Dippel DW, Bertina RM, et al Fibrinogen gamma' in ischemic stroke: a case-control study. Stroke. 2008;39: 1033–1035. doi: 10.1161/STROKEAHA.107.495499 18239174 10.1161/STROKEAHA.107.495499

[28] Drouet L, Paolucci F, Pasqualini N, Laprade M, Ripoll L, Mazoyer E, et al Plasma gamma'/gamma fibrinogen ratio, a marker of arterial thrombotic activity: a new potential cardiovascular risk factor? Blood Coagul Fibrinolysis. 1999;10: S35–39. 10070816

[29] Uitte de Willige SU, Pyle ME, Vos HL, de Visser MC, Lally C, Dowling NF, et al Fibrinogen gamma gene 3’-end polymorphisms and risk of venous thromboembolism in the African-American and Caucasian population. Thromb Haemost. 2009;101: 1078–1084. 19492150

[30] Bester J, Pretorius E. Effects of IL-1β, IL-6 and IL-8 on erythrocytes, platelets and clot viscoelasticity. Sci Rep. 2016;6: 32188 doi: 10.1038/srep32188 27561337 10.1038/srep32188 PMC4999875

[31] Machlus KR, Cardenas JC, Church FC, Wolberg AS. Causal relationship between hyperfibrinogenemia, thrombosis, and resistance to thrombolysis in mice. Blood. 2011;117: 4953–4963. doi: 10.1182/blood-2010-11-316885 21355090 10.1182/blood-2010-11-316885 PMC3100702

[32] Macrae FL, Domingues MM, Casini A, Ariëns RA, editors. The (Patho) physiology of Fibrinogen γ′. Semin Thromb Hemost. 2016;42: 344–355. doi: 10.1055/s-0036-1572353 27071047 10.1055/s-0036-1572353

[33] Pieters M, Kotze RC, Jerling JC, Kruger A, Ariëns RA. Evidence that fibrinogen γ′ regulates plasma clot structure and lysis and relationship to cardiovascular risk factors in black Africans. Blood. 2013;121: 3254–3260. doi: 10.1182/blood-2012-12-471482 23422752 10.1182/blood-2012-12-471482

[34] Undas A, Szułdrzynski K, Stepien E, Zalewski J, Godlewski J, Tracz W, et al Reduced clot permeability and susceptibility to lysis in patients with acute coronary syndrome: effects of inflammation and oxidative stress. Atherosclerosis. 2008;196: 551–557. doi: 10.1016/j.atherosclerosis.2007.05.028 17640649 10.1016/j.atherosclerosis.2007.05.028

[35] Chen Y-S, Torroni A, Excoffier L, Santachiara-Benerecetti AS, Wallace DC. Analysis of mtDNA variation in African populations reveals the most ancient of all human continent-specific haplogroups. Am J Hum Genet. 1995;57: 133–149. 7611282 PMC1801234

[36] Teo Y-Y, Small KS, Kwiatkowski DP. Methodological challenges of genome-wide association analysis in Africa. Nat Rev Genet. 2010;11: 149–160. doi: 10.1038/nrg2731 20084087 10.1038/nrg2731 PMC3769612

[37] Lammertyn L, Mels CM, Pieters M, Schutte AE, Schutte R. Ethnic-specific relationships between haemostatic and oxidative stress markers in black and white South Africans: The SABPA study. Clin Exp Hypertens. 2015;37: 511–517. doi: 10.3109/10641963.2015.1013123 25919704 10.3109/10641963.2015.1013123

[38] Pieters M, Vorster HH. Nutrition and hemostasis: a focus on urbanization in South Africa. Mol Nutr Food Res. 2008;52: 164–172. doi: 10.1002/mnfr.200700373 18080241 10.1002/mnfr.200700373

[39] Kotzé RC, Nienaber-Rousseau C, De Lange Z, De Maat MP, Hoekstra T, Pieters M. Genetic polymorphisms influencing total and gamma' fibrinogen levels and fibrin clot properties in Africans. Br J Haematol. 2015;168: 102–112. doi: 10.1111/bjh.13104 25156046 10.1111/bjh.13104

[40] Pieters M, De Maat MP, Jerling JC, Hoekstra T, Kruger A. Fibrinogen concentration and its role in CVD risk in black South Africans–effect of urbanisation. J Thromb Haemost. 2011;106: 448–456. 10.1160/TH11-03-0192 21800007

[41] Teo K, Chow CK, Vaz M, Rangarajan S, Yusuf S. The Prospective Urban Rural Epidemiology (PURE) study: examining the impact of societal influences on chronic noncommunicable diseases in low-, middle-, and high-income countries. Am Heart J. 2009;158: 1–7. doi: 10.1016/j.ahj.2009.04.019 19540385 10.1016/j.ahj.2009.04.019

[42] de Lange Z, Rijken DC, Hoekstra T, Conradie KR, Jerling JC, Pieters M. In black South Africans from rural and urban communities, the 4G/5G PAI-1 polymorphism influences PAI-1 activity, but not plasma clot lysis time. PloS one. 2013;8: e83151 doi: 10.1371/journal.pone.0083151 24386152 10.1371/journal.pone.0083151 PMC3875438

[43] Lisman T, de Groot PG, Meijers JC, Rosendaal FR. Reduced plasma fibrinolytic potential is a risk factor for venous thrombosis. Blood. 2005;105: 1102–1105. doi: 10.1182/blood-2004-08-3253 15466929 10.1182/blood-2004-08-3253

[44] Cook DG, Cappuccio FP, Atkinson RW, Wicks PD, Chitolie A, Nakandakare ER, et al Ethnic differences in fibrinogen levels: the role of environmental factors and the β-fibrinogen gene. Am J Epidemiol. 2001;153: 799–806. 11296154 10.1093/aje/153.8.799

[45] Lim BC, Ariëns RA, Carter AM, Weisel JW, Grant PJ. Genetic regulation of fibrin structure and function: complex gene-environment interactions may modulate vascular risk. Lancet. 2003;361: 1424–1431. doi: 10.1016/S0140-6736(03)13135-2 12727396 10.1016/S0140-6736(03)13135-2

[46] Mannila MN, Eriksson P, Ericsson C-G, Hamsten A, Silveira A. Epistatic and pleiotropic effects of polymorphisms in the fibrinogen and coagulation factor XIII genes on plasma fibrinogen concentration, fibrin gel structure and risk of myocardial infarction. Thromb Haemost. 2006;95: 420–427. doi: 10.1160/TH05-11-0777 16525568 10.1160/TH05-11-0777

[47] Reiner A, Carty C, Carlson C, Wan J, Rieder M, Smith J, et al Association between patterns of nucleotide variation across the three fibrinogen genes and plasma fibrinogen levels: the Coronary Artery Risk Development in Young Adults (CARDIA) study. J Thromb Haemost. 2006;4(6): 1279–1287. doi: 10.1111/j.1538-7836.2006.01907.x 16706972 10.1111/j.1538-7836.2006.01907.x

[48] Uitte de Willige S, de Visser MC, Houwing-Duistermaat JJ, Rosendaal FR, Vos HL, Bertina RM. Genetic variation in the fibrinogen gamma gene increases the risk for deep venous thrombosis by reducing plasma fibrinogen γ′ levels. Blood. 2005;106: 4176–4183. doi: 10.1182/blood-2005-05-2180 16144795 10.1182/blood-2005-05-2180

[49] van’t Hooft FM, von Bahr SJ, Silveira A, Iliadou A, Eriksson P, Hamsten A. Two common, functional polymorphisms in the promoter region of the β-fibrinogen gene contribute to regulation of plasma fibrinogen concentration. Arterioscler Thromb Vasc Biol. 1999;19: 3063–3070. 10591688 10.1161/01.atv.19.12.3063

[50] Yates A, Akanni W, Amode MR, Barrell D, Billis K, Carvalho-Silva D, et al Ensembl 2016. Nucleic Acids Res. 2016;44(D1): D710–D716. doi: 10.1093/nar/gkv1157 26687719 10.1093/nar/gkv1157 PMC4702834

[51] Barrett JC, Fry B, Maller J, Daly MJ. Haploview: analysis and visualization of LD and haplotype maps. Bioinformatics. 2005;21(2): 263–265. doi: 10.1093/bioinformatics/bth457 15297300 10.1093/bioinformatics/bth457

[52] Faul F, Erdfelder E, Lang A-G, Buchner A. G* Power 3: A flexible statistical power analysis program for the social, behavioral, and biomedical sciences. Behav Res Methods. 2007;39(2): 175–191. 17695343 10.3758/bf03193146

[53] Gabriel SB, Schaffner SF, Nguyen H, Moore JM, Roy J, Blumenstiel B, et al The structure of haplotype blocks in the human genome. Science. 2002;296: 2225–2229. doi: 10.1126/science.1069424 12029063 10.1126/science.1069424

[54] Dehghan A, Yang Q, Peters A, Basu S, Bis JC, Rudnicka AR, et al Association of novel genetic loci with circulating fibrinogen levels a genome-wide association study in 6 population-based cohorts. Circ Cardiovasc Genet. 2009;2: 125–133. doi: 10.1161/CIRCGENETICS.108.825224 20031576 10.1161/CIRCGENETICS.108.825224 PMC2764985

[55] Baumann RE, Henschen AH. Linkage disequilibrium relationships among four polymorphisms within the human fibrinogen gene cluster. Hum Genet. 1994;94: 165–170. 7913910 10.1007/BF00202863

[56] Behague I, Poirier O, Nicaud V, Evans A, Arveiler D, Luc G, et al β Fibrinogen gene polymorphisms are associated with plasma fibrinogen and coronary artery disease in patients with myocardial infarction: the ECTIM study. Circulation. 1996;93: 440–449. 8565160 10.1161/01.cir.93.3.440

[57] Thomas A, Lamlum H, Humphries S, Green F. Linkage disequilibrium across the fibrinogen locus as shown by five genetic polymorphisms, G/A-455 (HaeIII), C/T-148 (HindIII/AluI), T/G+1689 (AvaII), and BclI (beta-fibrinogen) and TaqI (alpha-fibrinogen), and their detection by PCR. Hum Mutat. 1994;3: 79–81. doi: 10.1002/humu.1380030117 7906988 10.1002/humu.1380030117

[58] Brown ET, Fuller GM. Detection of a complex that associates with the Bβ fibrinogen G− 455-A polymorphism. Blood. 1998;92: 3286–2193. 9787165

[59] Carty CL, Heagerty P, Heckbert SR, Jarvik GP, Lange LA, Cushman M, et al Interaction between fibrinogen and IL‐6 genetic variants and associations with cardiovascular disease risk in the cardiovascular health study. Ann Hum Genet. 2010;74: 1–10. doi: 10.1111/j.1469-1809.2009.00551.x 20059469 10.1111/j.1469-1809.2009.00551.x PMC2946374

[60] Danik JS, Paré G, Chasman DI, Zee RY, Kwiatkowski DJ, Parker A, et al Novel loci, including those related to crohn disease, psoriasis, and inflammation, identified in a genome-wide association study of fibrinogen in 17 686 women: the Women’s Genome Health Study. Circ Cardiovasc Genet. 2009;2: 134–141. doi: 10.1161/CIRCGENETICS.108.825273 20031577 10.1161/CIRCGENETICS.108.825273 PMC2749513

[61] de Vries PS, Chasman DI, Sabater-Lleal M, Chen MH, Huffman JE, Steri M, et al A meta-analysis of 120 246 individuals identifies 18 new loci for fibrinogen concentration. Hum Mol genet. 2016;25: 358–370. doi: 10.1093/hmg/ddv454 26561523 10.1093/hmg/ddv454 PMC4715256

[62] Kathiresan S, Yang Q, Larson MG, Camargo AL, Tofler GH, Hirschhorn JN, et al Common genetic variation in five thrombosis genes and relations to plasma hemostatic protein level and cardiovascular disease risk. Arterioscler Thromb Vasc Biol. 2006;26: 1405–1412. doi: 10.1161/01.ATV.0000222011.13026.25 16614319 10.1161/01.ATV.0000222011.13026.25

[63] Ken-Dror G, Humphries SE, Kumari M, Kivimaki M, Drenos F. A genetic instrument for Mendelian randomization of fibrinogen. Eur J Epidemiol. 2012;27: 267–279. doi: 10.1007/s10654-012-9666-x 22388766 10.1007/s10654-012-9666-x PMC4181528

[64] Klovaite J, Nordestgaard BG, Tybjærg-Hansen A, Benn M. Elevated fibrinogen levels are associated with risk of pulmonary embolism, but not with deep venous thrombosis. Am J Respir Crit Care Med. 2013;187: 286–293. doi: 10.1164/rccm.201207-1232OC 23220916 10.1164/rccm.201207-1232OC

[65] Van Der Bom JG, De Maat MP, Bots ML, Haverkate F, De Jong P, Hofman A, et al Elevated plasma fibrinogen cause or consequence of cardiovascular disease? Arterioscler Thromb Vasc Biol. 1998;18: 621–625. 9555868 10.1161/01.atv.18.4.621

[66] Wassel CL, Lange LA, Keating BJ, Taylor KC, Johnson AD, Palmer C, et al Association of genomic loci from a cardiovascular gene SNP array with fibrinogen levels in European Americans and African-Americans from six cohort studies: the Candidate Gene Association Resource (CARe). Blood. 2011;117: 268–275. doi: 10.1182/blood-2010-06-289546 20978265 10.1182/blood-2010-06-289546 PMC3037748

[67] Huffman J.E., de Vries P.S., Morrison A.C., Sabater-Lleal M., Kacprowski T., Auer P.L., et al Rare and low-frequency variants and their association with plasma levels of fibrinogen, FVII, FVIII, and vWF. Blood, 2015;126:e19–29. doi: 10.1182/blood-2015-02-624551 26105150 10.1182/blood-2015-02-624551 PMC4566813

[68] Undas A, Ariëns RA. Fibrin clot structure and function: a role in the pathophysiology of arterial and venous thromboembolic diseases. Arterioscler Thromb Vasc Biol. 2011;31(12): e88–99. doi: 10.1161/ATVBAHA.111.230631 21836064 10.1161/ATVBAHA.111.230631

[69] Jenkins GR, Seiffert D, Parmer RJ, Miles LA. Regulation of plasminogen gene expression by interleukin-6. Blood. 1997;89: 2394–2403. 9116283

[70] Kida M, Wakabayashi S, Ichinose A. Expression and induction by IL-6 of the normal and variant genes for human plasminogen. Biochem Biophys Res Commun. 1997;230: 129–132. doi: 10.1006/bbrc.1996.5909 9020027 10.1006/bbrc.1996.5909

[71] Mills JD, Ariëns RA, Mansfield MW, Grant PJ. Altered fibrin clot structure in the healthy relatives of patients with premature coronary artery disease. Circulation. 2002;106: 1938–1942. 12370216 10.1161/01.cir.0000033221.73082.06

[72] De Moerloose P, Boehlen F, Neerman-Arbez M, editors. Fibrinogen and the risk of thrombosis. Semin Thromb Hemost. 2010;36: 7–17. doi: 10.1055/s-0030-1248720 20391292 10.1055/s-0030-1248720

[73] Zerbino DR, Johnson N, Juetteman T, Sheppard D, Wilder SP, Lavidas I, et al Ensembl regulation resources. Database (Oxford). 2016; doi: 10.1093/database/bav119 26888907 10.1093/database/bav119 PMC4756621

