# Supplementary material for: Fibrinogen and clot-related phenotypes determined by fibrinogen polymorphisms: Independent and IL-6-interactive associations
Source: PLoS One. 2017 Nov 3;12(11):e0187712. doi: 10.1371/journal.pone.0187712 (PMC5669433; doi:10.1371/journal.pone.0187712)
Supplement: S1 Table — (DOCX) [file pone.0187712.s001.docx]

S1 Table. Primer and synthetic control sequences used for KASP analyses

| Assay ID | Primer_AlleleFAM^#^ | | Primer_AlleleHEX^#^ | | Primer_Common^*^ |
| --- | --- | --- | --- | --- | --- |
| *FGB*-rs1800790 | CATTATGATATAACATTACTATTGATTTTAATA | **A** | CATTATGATATAACATTACTATTGATTTTAATG | **G** | ACAATGACATAATTCTATTTCAAAAGGGGC |
| *FGB*-rs1800789 | TCCTGTATATATTTTAATTAATAGCCACATAA | **A** | CCTGTATATATTTTAATTAATAGCCACATAG | **G** | GGTTTTTAAAGGGATACATGATCTGACAGA |
| *FGB*-rs7439150 | CAGCTAATATGAAGAACACTGCACTA | **A** | CAGCTAATATGAAGAACACTGCACTG | **G** | GGGAAAGGGAGTACTATTTGTTCAGTTAT |
| *FGB*-rs4463047 | CTTGGTTCATAAATAAAGAGGAACTAGAA | **T** | CTTGGTTCATAAATAAAGAGGAACTAGAG | **C** | CTATAATGGATGTATGCACTTGGGACATA |
| Control  *FGB*-rs1800790 | Major_5’TCATAGAATAGGGTATGAATTTGTTATTTTGTTATTTTGATTAATGTCTAAAACAAAAGATAAACA*CATTATGATATAACATTACTATTGATTTTAAT****[G]***GCCCCTTTTGAAATAGAATTATGTCATTGTCAGAAAACATAAGCATTTATGGTATATCATTAATGAGTCACGATTTTAGTGGTTGCCTTGTGAGTAG’3  Minor_5’TCATAGAATAGGGTATGAATTTGTTATTTTGTTATTTTGATTAATGTCTAAAACAAAAGATAAACA*CATTATGATATAACATTACTATTGATTTTAAT****[A]***GCCCCTTTTGAAATAGAATTATGTCATTGTCAGAAAACATAAGCATTTATGGTATATCATTAATGAGTCACGATTTTAGTGGTTGCCTTGTGAGTAG’3 | | | | |
| Control  *FGB*-rs1800789 | Major_5’TGTTATCAGTTATATTTTCATGGAAAATAT*TCCTGTATATATTTTAATTAATAGCCACATA****[G]***ATATTTGCTTTTTCTGTCAGATCATGTATCCCTTTAAAAACCATTAAAGCTAAAGAAAAAAATCTTAAAATATAATATTTACTCATTGCTAATAAAGGGAACACAAATTTGGAAAGACTCAAGTGAATATTTTT’3  Minor_5’TGTTATCAGTTATATTTTCATGGAAAATAT*TCCTGTATATATTTTAATTAATAGCCACATA****[A]***ATATTTGCTTTTTCTGTCAGATCATGTATCCCTTTAAAAACCATTAAAGCTAAAGAAAAAAATCTTAAAATATAATATTTACTCATTGCTAATAAAGGGAACACAAATTTGGAAAGACTCAAGTGAATATTTTT’3 | | | | |
| Control  *FGB*-rs7439150 | Major_5’TCAAGCCAGAGAGCTGCTGGGCAGGACAGATTATCTCTGTTTTCTCTGTCACTGATTTACTACCCTTGACTTATGCAATGACAT*CAGCTAATATGAAGAACACTGCACT****[G]***ATGACCTCAAAATAACTGAACAAATAGTACTCCCTTTCCCCTCCACCAATGGCATCAACATTCTAAAAAAATTTAAGCATCTTAAT’3  Minor_5’TCAAGCCAGAGAGCTGCTGGGCAGGACAGATTATCTCTGTTTTCTCTGTCACTGATTTACTACCCTTGACTTATGCAATGACAT*CAGCTAATATGAAGAACACTGCACT****[A]***ATGACCTCAAAATAACTGAACAAATAGTACTCCCTTTCCCCTCCACCAATGGCATCAACATTCTAAAAAAATTTAAGCATCTTAAT’3 | | | | |
| Control  *FGB*-rs4463047 | Major_5’ATACATATATATATATACACATATATAATTATATATATGTATATATAATTATACAATTAGGACTGAAAGTCTGCAACAAACTATAATGGATGTATGCACTTGGGACATAAGC***[T]****TCTAGTTCCTCTTTATTTATGAACCAAG*AGAAACAGCTAACTCAGGGATTGTTTTTAAACTGACTACAGATTCCCACCATGGG’3  Minor_5’ATACATATATATATATACACATATATAATTATATATATGTATATATAATTATACAATTAGGACTGAAAGTCTGCAACAAACTATAATGGATGTATGCACTTGGGACATAAGC***[C]****TCTAGTTCCTCTTTATTTATGAACCAAG*AGAAACAGCTAACTCAGGGATTGTTTTTAAACTGACTACAGATTCCCACCATGGG’3 | | | | |

FGB = fibrinogen beta chain gene; ^*^ Underlined sequences correspond to the common primer annealing position; ^#^ Italic sequences correspond to the discriminating primer annealing position; Heterozygote control comprised 50/50 major/minor mixture.
