# Supplementary material for: Fibrinogen and clot-related phenotypes determined by fibrinogen polymorphisms: Independent and IL-6-interactive associations
Source: PLoS One. 2017 Nov 3;12(11):e0187712. doi: 10.1371/journal.pone.0187712 (PMC5669433; doi:10.1371/journal.pone.0187712)
Supplement: S2 Table — (DOCX) [file pone.0187712.s002.docx]

**S2 Table. Basic descriptive characteristics of the study population as published by Kotzé *et al,* (2015)**

| **Variable** | **Total population (n = 2010)** |
| --- | --- |
| Age (years) | 48.3 (41.9-56.4) |
| HIV positive (%) | 16.2 |
| Blood pressure (mmHg)  Systolic  Diastolic | 134 ± 24.5  87.7 ± 14.5 |
| Body mass index (kg/m^2^) | 23.0 (19.3-28.9) |
| Waist circumference (cm) | 77.5 (70.2-87.7) |
| HbA1c (%) | 5.50 (5.30-5.80) |
| Serum total cholesterol (mM) | 5.01 ± 1.38 |
| Serum HDL-cholesterol (mM) | 1.52 ± 0.63 |
| Serum LDL-cholesterol (mM) | 2.92 ± 1.17 |
| Serum triglycerides (mM) | 1.08 (0.82-1.55) |
| Serum IL-6 (pg/ml) | 2.84 (0.75-5.76) |
| Serum CRP (mg/l) | 3.29 (0.96-9.34) |
| Plasma homocysteine (μM) | 9.18 (7.45-12.1) |
| Plasma fibrinogen (g/l) | 2.90 (2.30-5.00) |
| Plasma fibrinogen γ’ (g/l) | 0.31 (0.23-0.45) |
| γ’ ratio (%) | 10.2 (7.14-14.6) |
| Lag time (min) | 6.46 ± 1.97 |
| Slope (x10^−3^ au/s) | 8.91 (6.48-12.0) |
| Maximum absorbance (nm) | 0.43 ± 0.16 |
| Clot lysis time (min) | 57.3 ± 11.2 |

Normally distributed data reported as: mean ± SD and non-parametric data as: median (25^th^-75^th^ percentile). CRP, C-reactive protein; HbA1_c_, glycosylated haemoglobin; HIV, human immunodeficiency virus; HDL-cholesterol, high-density lipoprotein cholesterol; IL-6, Interleukin-6; LDL-cholesterol, low-density lipoprotein cholesterol; γ’, gamma prime.
