# Supplementary material for: Fibrinogen and clot-related phenotypes determined by fibrinogen polymorphisms: Independent and IL-6-interactive associations
Source: PLoS One. 2017 Nov 3;12(11):e0187712. doi: 10.1371/journal.pone.0187712 (PMC5669433; doi:10.1371/journal.pone.0187712)
Supplement: S3 Table — (DOCX) [file pone.0187712.s003.docx]

**S3 Table. Associations of individual SNPs with fibrinogen γ′, % γ′ and total fibrinogen as published by Kotzé *et al,* (2015)**

| **Genotype** | **Fibrinogen γ' (g/l)** | **γ' ratio** | **Fibrinogen (g/l)** |
| --- | --- | --- | --- |
| rs2070011 |  |  |  |
| GG | 0.30 (0.23-0.44) *  (n = 1021) | 10.1 (7.14-14.7)  (n = 987) | 2.80 (2.30-5.00)  (n = 1112) |
| GA | 0.32 (0.23-0.46) ^0^  (n = 428) | 10.3 (7.42-15.0)  (n = 405) | 2.90 (2.20-5.00)  (n = 422) |
| AA | 0.39 (0.33-0.55) * ^0^  (n = 40) | 11.1 (6.55-17.6)  (n = 36) | 3.70 (2.80-6.20)  (n = 37) |
| p-value | 0.008 | 0.50 | 0.11 |
| p-value after adjustment for fibrinogen γ’ | - | 0.52 | 0.52 |
| rs1049636 |  |  |  |
| TT | 0.31 (0.23-0.45)  (n = 1058) | 10.3 (7.22-14.8)  (n = 1020) | 2.80 (2.20-4.50) *  (n = 1069) |
| TC | 0.31 (0.23-0.47)  (n = 392) | 10.3 (7.05-15.1)  (n = 371) | 3.00 (2.20-5.30) ^0^  (n = 383) |
| CC | 0.32 (0.22-0.57)  (n = 45) | 8.30 (5.67-13.6)  (n = 43) | 5.00 (2.80-6.25) * ^0^  (n = 44) |
| p-value | 0.61 | 0.08 | 0.0009 |
| p-value after adjustment for fibrinogen | 0.93 | 0.93 | - |
| rs1800791 |  |  |  |
| GG | 0.31 (0.23-0.45)  (n = 1462) | 10.2 (7.21-14.5)  (n = 1402) | 2.90 (2.20-5.00) *  (n = 1460) |
| GA | 0.31(0.23-0.46)  (n = 274) | 10.3 (7.11-15.1)  (n = 265) | 3.00 (2.30-5.00)  (n = 286) |
| AA | 0.35 (0.28-0.50)  (n = 11) | 6.97 (5.39-10.2)  (n = 11) | 5.30 (2.90-6.40) *  (n = 11) |
| p-value | 0.21 | 0.40 | 0.04 |
| p-value after adjustment for fibrinogen | 0.48 | 0.48 | - |

Data reported as median (25^th^-75^th^ percentile). * ^0^ Means with the same symbol differ significantly. A, adenine; C, cytosine; G, guanine; SNP, single nucleotide polymorphism; T, thymine; γ’, gamma prime.
