# Supplementary material for: Fibrinogen and clot-related phenotypes determined by fibrinogen polymorphisms: Independent and IL-6-interactive associations
Source: PLoS One. 2017 Nov 3;12(11):e0187712. doi: 10.1371/journal.pone.0187712 (PMC5669433; doi:10.1371/journal.pone.0187712)
Supplement: S4 Table — (DOCX) [file pone.0187712.s004.docx]

**S4 Table. Associations of individual SNPs with clot-related phenotypes as published by Kotzé *et al,* (2015)**

| **Genotype** | **Clot lysis time (minutes)** | **Lagtime (minutes)** | **Slope** | **Maximum absorbance** |
| --- | --- | --- | --- | --- |
| rs1800787 |  |  |  |  |
| CC | 57.3 [56.7-57.9]  (n = 1317) | 6.36 [6.25-6.47]  (n = 1345) | 9.83 [9.58-10.1]  (n = 1316) | 0.43 [0.42-0.44] *  (n = 1318) |
| CT | 56.2 [54.3-58.0]  (n = 165) | 6.53 [6.23-6.83]  (n = 170) | 9.93 [9.30-10.6]  (n = 168) | 0.47 [0.44-0.50] *  (n = 165) |
| TT | 57.3 [49.8-64.7]  (n = 9) | 7.06 [5.51-8.60]  (n = 9) | 9.76 [8.00-11.5]  (n = 9) | 0.48 [0.42-0.54]  (n = 9) |
| p-value | 0.27 | 0.16 | 0.82 | 0.001 |
| rs2070011 |  |  |  |  |
| GG | 57.4 [56.7-58.1]  (n = 1034) | 6.43 [6.30-6.55]  (n = 1061) | 9.69 [9.41-9.96]  (n = 1038) | 0.43 [0.42-0.43] *  (n = 1035) |
| GA | 56.9 [55.9-57.9]  (n = 440) | 6.29 [6.11-6.47]  (n = 446) | 10.2 [9.79-10.7]  (n = 438) | 0.45 [0.43-0.46] *  (n = 440) |
| AA | 59.1 [55.2-63.0]  (n = 43) | 6.91 [6.34-7.48]  (n = 42) | 9.11 [7.88-10.3]  (n = 42) | 0.45 [0.41-0.50]  (n = 43) |
| p-value | 0.93 | 0.90 | 0.22 | 0.01 |
| p-value adjusted for fibrinogen γ’ | 0.97 | 0.81 | 0.21 | 0.06 |
| rs1049636 |  |  |  |  |
| TT | 57.3 [56.7-58.0]  (n = 1082) | 6.37 [6.25-6.48]  (n = 1106) | 9.76 [9.49-10.0]  (n = 1081) | 0.43 [0.42-0.44] *  (n = 1083) |
| TC | 57.2 [56.0-58.3]  (n = 394) | 6.47 [6.28-6.67]  (n = 401) | 9.93 [9.48-10.4]  (n = 395) | 0.44 [0.43-0.46]  (n = 394) |
| CC | 57.7 [54.3-61.0]  (n = 46) | 6.58 [6.09-7.08]  (n = 48) | 10.1 [8.74-11.5]  (n = 48) | 0.48 [0.43-0.53] *  (n = 46) |
| p-value | 0.94 | 0.27 | 0.42 | 0.01 |
| p-value adjusted for fibrinogen | 0.88 | 0.84 | 0.72 | 0.33 |
| rs5985 |  |  |  |  |
| CC | 57.0 [56.3-57.7]  (n = 1105) | 6.31 [6.20-6.42] *  (n = 1130) | 9.91 [9.65-10.2]  (n = 1110) | 0.43 [0.42-0.44]  (n = 1105) |
| CA | 57.8 [56.6-58.9]  (n = 360) | 6.61 [6.39-6.84] *  (n = 366) | 9.74 [9.23-10.2]  (n = 357) | 0.44 [0.42-0.45]  (n = 361) |
| AA | 58.8 [54.9-62.7]  (n = 28) | 6.22 [5.61-6.83]  (n = 30) | 8.67 [7.45-9.90]  (n = 28) | 0.40 [0.32-0.48]  (n = 28) |
| p-value | 0.39 | 0.04 | 0.30 | 0.55 |

Data reported as mean [95% confidence interval]. * Means with the same symbol differ significantly. A, adenine; C, cytosine; G, guanine; SNP, single nucleotide polymorphism; T, thymine; γ’, gamma prime.
